# Polymer-Bonded/Bolted Steel Plates Versus UHPFRC Overlay for Controlling Deflection in RC Shallow Beams with Planted Columns—Experimental Insights

**DOI:** 10.3390/polym17223051

**Published:** 2025-11-18

**Authors:** Hussein Elsanadedy, Abdulaziz Baatiah, Aref Abadel, Husain Abbas, Tarek Almusallam, Yousef Al-Salloum

**Affiliations:** Chair of Research and Studies in Strengthening and Rehabilitation of Structures, Department of Civil Engineering, College of Engineering, King Saud University, P.O. Box 800, Riyadh 11421, Saudi Arabia; helsanadedy@ksu.edu.sa (H.E.); 447105717@student.ksu.edu.sa (A.B.); aabadel@ksu.edu.sa (A.A.); habbas@ksu.edu.sa (H.A.); musallam@ksu.edu.sa (T.A.)

**Keywords:** RC wide beams, planted columns, deflection control, steel plates, UHPFRC layer, flexural strengthening

## Abstract

Reinforced concrete (RC) joist slabs are common in Middle Eastern buildings, where architectural needs often necessitate planting columns on shallow beams. Although such beams typically satisfy flexural and shear design requirements, their serviceability is frequently compromised by excessive deflections. This study experimentally investigated the effectiveness of polymer-bonded/bolted steel plates versus an Ultra-High-Performance Fiber-Reinforced Concrete (UHPFRC) overlay, applied to the compression face, in controlling the deflection of shallow beams with planted columns. Four half-scale beams were tested under single-point loading, including two unstrengthened specimens to be used as reference beams. The first control beam reflected typical design practice—adequate in strength but exceeding code deflection limits—while the second specimen was designed to achieve similar flexural capacity with serviceable deflection. The remaining two beams were externally strengthened using either steel plates or UHPFRC overlay. Experimental results were analyzed in terms of failure mode, peak load, and deflection response. Both strengthening methods improved bending performance, stiffness, and load capacity, with UHPFRC showing superior effectiveness. Simplified analytical equations provided reasonable predictions of deflection and ultimate load. The findings highlight the potential of compression-side strengthening, particularly using UHPFRC, to enhance the serviceability of shallow RC beams supporting planted columns.

## 1. Introduction

RC joist slabs (also known as Hordy slabs) are among the most prevalent floor systems in buildings in the Middle East. This system comprises RC shallow beams, joists, and a topping concrete overlay [1]. RC shallow beams are generally categorized by a width/depth ratio exceeding two and a maximum depth of 350 mm [2]. The popularity of this design stems from its numerous constructional and architectural benefits, including the ability to maintain a shallow structural depth and its ease of formwork and reinforcement placement, which may contribute to overall project cost efficiency.

Shallow beams are particularly advantageous when there are limitations on structural depth. However, architectural considerations frequently require the placement of planted columns on such shallow beams. The planted columns transfer loads from upper floors to the shallow beams, which subsequently distribute them to the supporting columns [3]. The practice of constructing shallow beams with planted columns is common in Saudi Arabia, prompting a closer investigation into the structural behavior of these configurations.

While planting columns on shallow beams is practically needed, it may also present significant challenges, if not carefully designed and executed. These challenges range from serviceability issues, such as excessive deflection, to potentially severe consequences, including structural failure. Particularly, many concrete buildings in the region that incorporate planted columns are now considered structurally inadequate by current standards [1]. To address these issues, it is crucial to strengthen the shallow beams supporting planted columns, thereby mitigating the associated risks.

Steel plates are widely utilized for strengthening reinforced concrete beams, effectively enhancing both flexural and shear capacities. This method is widely favored due to several advantages, including the availability and cost-effectiveness of steel plates, their uniform material properties (isotropic), ease of handling, high ductility, and excellent fatigue strength. Owing to these advantages, steel plate reinforcement serves as a competent means of strengthening RC beams [4]. Bonding plates to the tension zones of beams represents the most widely used method of plating. Placing the plate at this location maximizes its distance from the compression zone, thereby optimizing the composite flexural action and enhancing the beam’s overall structural performance [4,5]. In the present investigation, the primary objective is to reduce deflection rather than to enhance flexural capacity. Accordingly, steel plates were installed on the compression face of the specimen. This approach aimed to reduce deflection (instantaneous as well as long-term) [6].

To date, no experimental studies have been found focusing on the upgrading of shallow beams utilizing steel plates. However, several researchers have examined the employment of steel plates for increasing the bending capacity of conventional beams, characterized by a depth-to-width ratio greater than 1.0. Alfeehan [7] explored the retrofitting of beams through bolted steel plates. A total of 4 beam specimens, each measuring 100 mm in width, 150 mm in depth, and 1500 mm in length, were tested in the event of two-point flexure up to failure. One beam was a reference specimen, while the other three beams were externally upgraded on the tension face with steel plates having thickness varying from 0.5 mm to 1.5 mm. The findings indicated an upgrade in failure load varying from 48% to 88%. Similarly, Aykac et al. [8] tested 13 beams, each having section of 200 × 500 mm and length of 4500 mm. One unstrengthened specimen was tested alongside twelve beams strengthened with tension-face steel plates varying in thickness from 1.5 mm to 6 mm, all anchored or bolted in place. The strengthened specimens demonstrated an upgrade in peak load varying from 12% to 100%. Additional studies have also confirmed that applying steel plates to the tension region of beams is an efficient technique for improving their flexural performance [9,10,11,12,13]. Recent research has continued to evolve these methods, exploring novel prestressing techniques using external bolts to address specific deficiencies like substandard tension lap splices, as well as hybrid systems that combine steel plates with CFRP [14,15].

Shatha et al. [16,17] examined the bending response of beams retrofitted with steel plates installed on the compression face. Four full-scale beams, each measuring 200 × 300 × 2400 mm, were tested in the event of 4-point flexure. The experimental campaign had one control un-retrofitted specimen and three specimens retrofitted with steel plates of different thicknesses. The findings demonstrated that the bending resistance of the retrofitted beams increased by 73% to 149% relative to the un-retrofitted specimen. Furthermore, the addition of compression-face steel plates altered the failure mechanism from brittle, as observed in the control beam, to ductile behavior, with the mode of failure varying according to the plate thickness.

Another retrofitting material utilized in the present study is a UHPFRC, cementitious composite material developed for enhancing the performance of RC members. UHPFRC is composed of cement, water, fine aggregates such as sand, nano-scale materials like silica fume, admixtures (superplasticizers), and steel fibers [18,19]. UHPFRC provides substantial advantages for structural strengthening applications owing to its exceptionally high compressive strength, along with significantly improved tensile and flexural performance attributed to the incorporation of steel fibers. Its dense microstructure greatly improves durability, reduces porosity and increases resistance to environmental conditions. Despite higher initial costs, UHPFRC’s longevity and reduced maintenance needs offer long-term economic benefits, thus being optimum for applications that need durability and superior strength [20]. Owing to its outstanding mechanical characteristics and long-term durability, UHPFRC has emerged as a commonly utilized material for retrofitting existing RC beams. The superior properties of UHPFRC make it especially effective in upgrading the ultimate load and prolonging the service life of structural elements in need of strengthening [21,22].

In research by Paschalis et al. [23], an experimental campaign explored the UHPFRC efficacy in retrofitting beams by applying it to the tension side. Six beams, each measuring 150 × 300 × 2200 mm, were tested under 4-point flexure. Two beams were unstrengthened, while the remaining four specimens were retrofitted with a UHPFRC layer (thickness = 50 mm, compressive strength = 136.5 MPa), both without and with internal steel bars. The findings demonstrated that the UHPFRC layer notably enhanced beam stiffness and delayed the onset of cracking. Even though the upgrade in peak load was modest for beams retrofitted with UHPFRC alone, the inclusion of steel bars within the UHPFRC led to a significant improvement, with a maximum load capacity increase of 87% with respect to the reference beams.

Elsanadedy et al. [24] examined the bending response of beams retrofitted using a hybrid technique that combined a UHPC (Ultra-High-Performance Concrete) layer and NSM (near-surface mounted) pultruded CFRP strips applied to the compression region. The experimental results indicated a remarkable 107% increase in flexural capacity relative to the deficient control beams. Additionally, the hybrid system significantly improved ductility and energy dissipation while providing a well-balanced enhancement in stiffness and mitigating premature failure mechanisms.

Yin et al. [25] carried out a testing investigation to assess the response of slabs upgraded with UHPFRC. A total of five specimens were subjected to 3-point bending tests, comprising one un-retrofitted reference specimen and four specimens retrofitted with UHPFRC layers of different thicknesses (ranging from 25 mm to 50 mm, compressive strength = 150 MPa), applied on the tension side with and without embedded reinforcement. The findings demonstrated that the UHPFRC overlay substantially improved the stiffness of the beams and delayed the onset of cracking. The incorporation of reinforcement within the UHPFRC layer additionally enhanced the peak load. Nevertheless, ensuring proper cover was found to be critical for achieving effective bonding and optimal performance. Overall, the use of UHPFRC on the tension face of flexural members—whether cast in place or prefabricated, with or without additional reinforcement—was effective for enhancing both bending resistance and stiffness [26,27,28,29,30,31]. This effectiveness, however, is critically dependent on the behavior of the UHPFRC-concrete interface, which governs the composite response. To enhance this composite action and further boost capacity, recent studies have also investigated incorporating internal reinforcement, such as GFRP rebars, and mechanical anchorage within the UHPFRC overlay [32,33].

RC shallow beams with planted columns are prevalent in existing buildings in the non-seismic regions of the Middle East. However, numerous construction projects have demonstrated that, although these beams are designed to satisfy flexural and shear requirements, their deflections under service loads frequently exceed the limits prescribed by applicable codes. In a recent study by the authors, Baatiah et al. [34] experimentally investigated, for the first time, the effectiveness of utilizing CFRP (carbon-fiber-reinforced polymer) sheets alone versus a hybrid system combining CFRP sheets and CFRP/honeycomb plates to control the deflection of RC shallow beams with planted columns. In this regard, 4 half-scale beams were tested to failure, including two control specimens: one representing conventional practice with excessive deflection and another designed to satisfy code-based limits. The other two beams were strengthened on the tension side using different techniques—one with externally bonded CFRP sheets and the other with the hybrid system. The strengthened specimens exhibited significant improvements in flexural performance, achieving 65–71% higher peak loads, 63–67% greater stiffness, and 45–48% lower deflections, thus meeting serviceability requirements. However, both strengthening systems reduced deflection ductility by approximately 57% and 33%, respectively, when compared with the code-based control specimen. This was due to intermediate crack-induced debonding of the tension-side strengthening layers.

Even though the strengthening systems examined by the authors in Ref. [34] successfully controlled the deflection in RC shallow beams with planted columns, the deflection ductility of the strengthened beams was significantly reduced. Moreover, in some practical situations, the bottom tension surface of shallow beams may not be accessible for applying strengthening layers. Accordingly, in this study, the authors conducted a second phase of research to examine two new strengthening schemes applied to the compression side of shallow beams with planted columns, aiming to enhance their flexural stiffness and thereby reduce mid-span deflections to within the permissible limits specified by design codes. In this aspect, polymer-bonded/bolted steel plates and a UHPFRC layer were added on the compression side for strengthening of shallow beams. Four large-scale specimens with mid-span planted columns were tested to failure: two controls, one exceeding and one meeting code deflection limits, and two externally strengthened with steel plates and a UHPFRC layer added on the compression side.

## 2. Testing Framework

### 2.1. Experimental Matrix

The experimental program comprised flexural testing of 4 half-scale shallow beams, each featuring a mid-span planted column. Table 1 outlines the experimental matrix, while Table 2 presents a comparison between the measured total deflections and the allowable limits specified by codes [35,36]. The reported deflections account for both immediate responses to live loads and additional long-term deflections resulting from dead loads and sustained live loads. In this study, the sustained live load was assumed to represent 25% of the total live load typically considered in residential building design.

**Figure 1:**
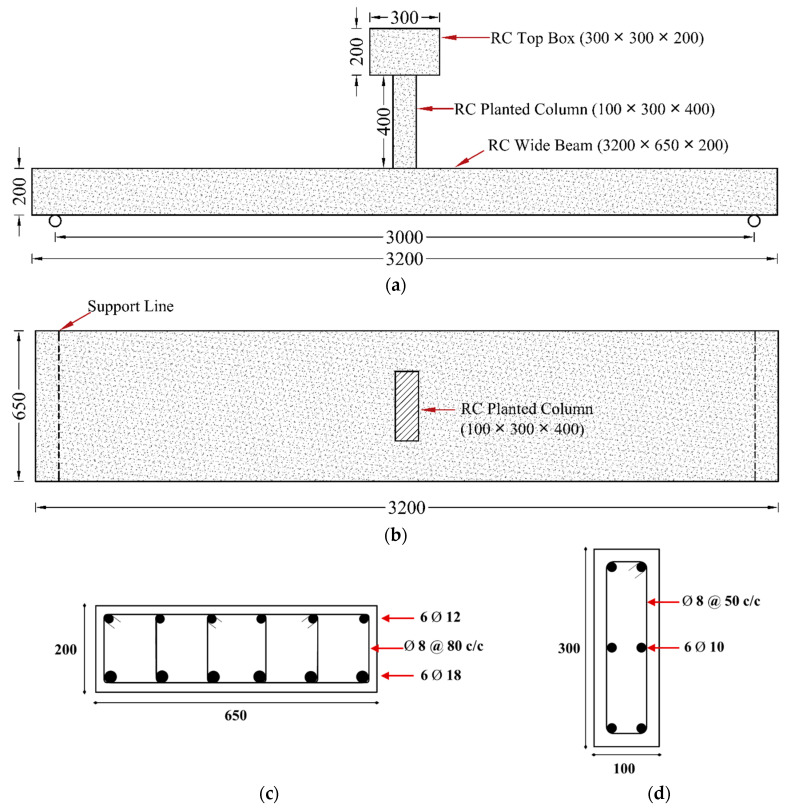
Details of reference beam CON-AB: (**a**) Elevation view; (**b**) Top view; (**c**) Beam section; (**d**) Column section (Units: mm).

The first reference specimen, designated CON-AB, was constructed as a half-scale model representative of shallow beams commonly found in existing structures (see Figure 1). In this specimen, the concrete dimensions were scaled down by half; nevertheless, the reinforcement was designed to maintain the same longitudinal and transverse steel reinforcement ratios as those used in the prototype shallow beams. The size of steel rebars in the test specimen was selected to be similar to that used in the typical construction practice in existing buildings. It should be mentioned that the scale used in the test specimens has no effect on the experimental results. Since the primary objective of this study is a comparative assessment of different strengthening techniques against control specimens, the conclusions drawn from the relative performance (e.g., percentage increases in stiffness and strength) remain valid and meaningful. The control specimen CON-AB featured a rectangular cross-section measuring 650 mm × 200 mm, with an overall length of 3200 mm and a clear span of 3000 mm. At mid-span, a planted RC column was incorporated, sized 100 × 300 × 400 mm (width × depth × height), and capped with a box measuring 300 × 300 × 200 mm. The beam was reinforced with 6 ϕ 18 mm tension bars and 6 ϕ 12 mm compression bars, along with ϕ8 mm stirrups spaced at 80 mm intervals. The column reinforcement comprised 6 ϕ 10 mm longitudinal bars and ϕ8 mm transverse ties spaced at 50 mm, while the top box was heavily reinforced to prevent localized failure. According to Table 1, CON-AB satisfied both flexural and shear design criteria [35,36]; however, Table 2 reveals that its total deflection exceeded the limits prescribed by relevant design codes.

**Figure 2:**
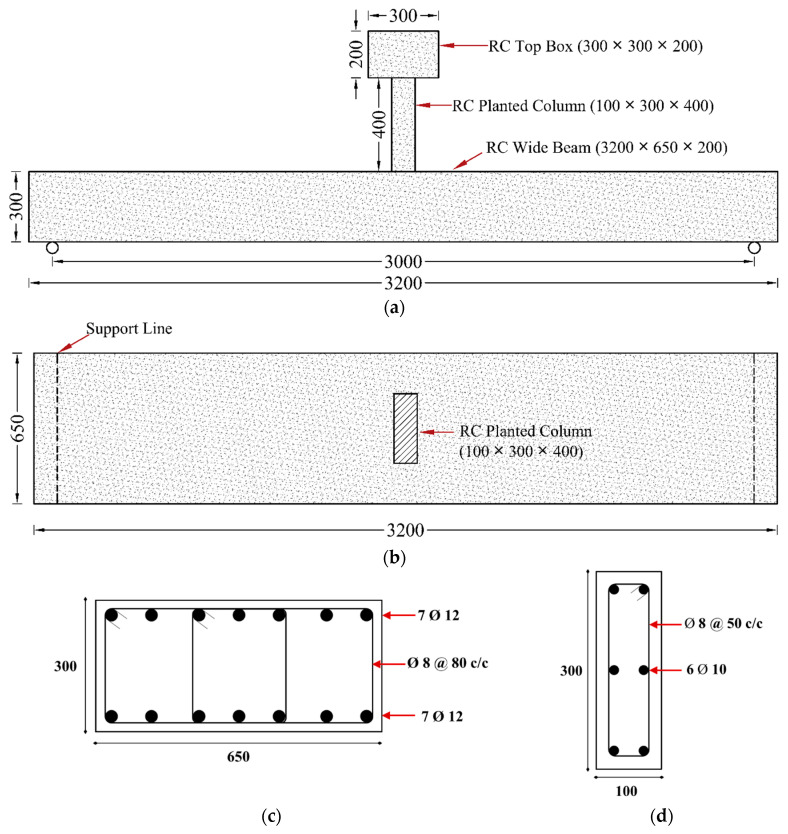
Details of reference beam CON-ACI: (**a**) Elevation view; (**b**) Top view; (**c**) Section of shallow beam; (**d**) Section of planted column (Units: mm).

The second reference specimen, named CON-ACI, was prepared to replicate the flexural capacity of the reference beam CON-AB while enhancing stiffness to comply with deflection limits specified by design codes, as outlined in Table 1 and Table 2. Figure 2 illustrates the configuration of CON-ACI. Relative to CON-AB, this specimen featured an increased overall depth of 300 mm, 7 ϕ 12 mm longitudinal bars in both the tension and compression zones, and a reduced stirrup arrangement consisting of four branches. All other design parameters remained unchanged.

**Figure 3:**
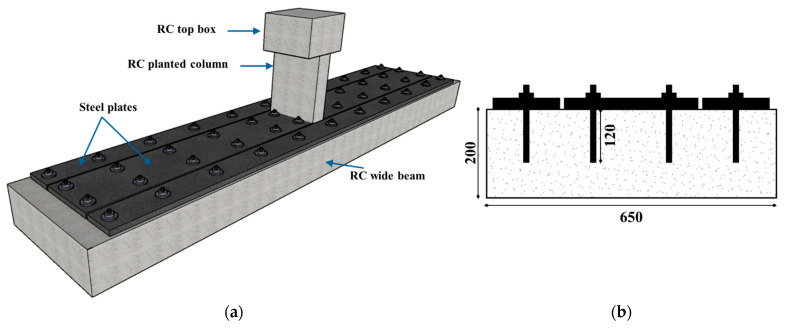

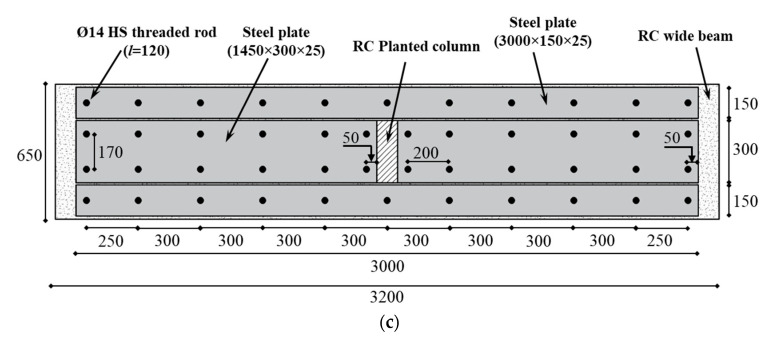
Details of upgraded beam S-SP: (**a**) 3D view; (**b**) Shallow beam section (**c**) Top view (Units: mm).

The last two beams, S-SP and S-UHC, were upgraded specimens designed to increase the flexural stiffness of CON-AB to a level comparable with CON-ACI. Both retained the same concrete dimensions and steel reinforcement as reference beam CON-AB. Figure 3 shows the S-SP upgrading scheme, which employed externally bonded and bolted steel plates on the compression side. The 25 mm-thick plates were secured with polymer adhesive (SIKA 31/41) and 14 mm-diameter, 120 mm-long high-strength threaded steel rods.

**Figure 4:**
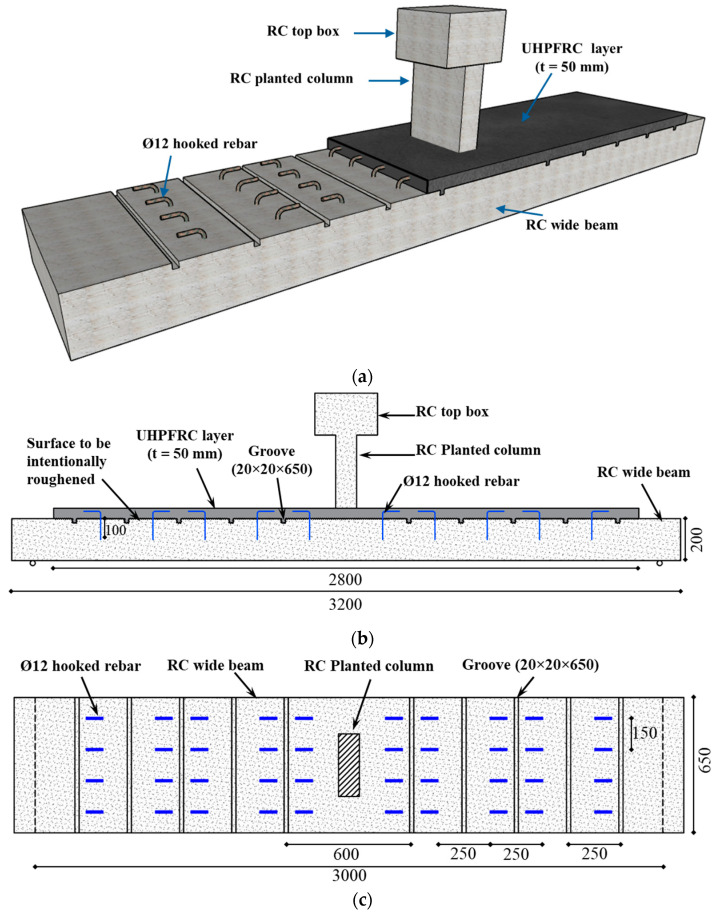
Details of upgraded beam S-UHC: (**a**) 3D view; (**b**) Side view; (**c**) Plan view showing the grooves and hooked rebars (Units: mm).

Figure 4 presents the second strengthening configuration for specimen S-UHC. As illustrated, the beam was strengthened on the compression face with a UHPFRC layer of 50-mm thickness. This overlay was cast atop the RC beam. To improve the bond between the original surface and the UHPFRC overlay, 12 mm-diameter steel bars were anchored within the beam and extended into the UHPFRC layer through hooked terminations, as depicted in Figure 4.

The thicknesses of the steel plates and UHPFRC utilized for the retrofitting of beams S-SP and S-UHC were carefully designed to enhance the stiffness of the control beam CON-AB to achieve equivalence with the stiffness of the reference beam CON-ACI. This design process adhered to the analytical procedure discussed later in Section 4. Moreover, the anchorage systems employed in both strengthened specimens were specifically designed to resist the shear stresses developed along the interaction between the original surface and the applied retrofitting material. This ensures an efficient transfer of forces and prevents premature failure at the bond interface. The spacing of the anchoring rods was determined using the following formula.

(1) $$s=\frac{n\;F_{r}}{\tau }$$

where n is the number of rod rows; $F_{r}$ is the shear capacity of the rod (=*A_r_f_y_* where *A_r_* is the rod area, and *f_y_* is the rod yield stress); and τ is the shear flow at the interface, given by

(2) $$\tau =\frac{Q\;\eta }{I_{x}}$$

where Q is the peak shear force in the strengthened specimen; η is the static moment of area of the strengthening layer about the *x*-axis of the transformed section of retrofitted specimen; and $I_{x}$ is the second moment of area of strengthened specimen.

### 2.2. Materials Properties

To assess the mechanical properties of the concrete, compression tests were conducted in compliance with Ref. [37]. On the day of testing, the average compressive strength of three standard cylinders measuring 150 mm by 300 mm was 40 MPa. Tensile testing was used to determine the mechanical properties of the reinforcing steel bars (AlRajhi, Riyadh, Saudi Arabia). Standard tension tests were conducted for each bar diameter in accordance with Ref. [38]. Table 3 reports the steel bars’ measured mechanical properties.

**Figure 5:**
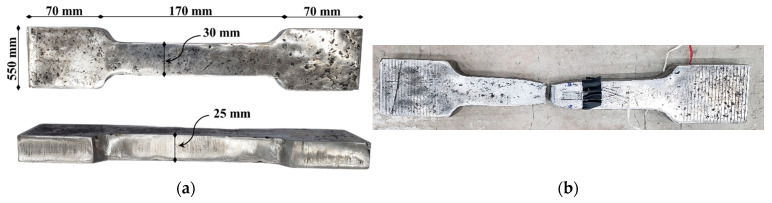
Testing of steel plate coupons: (**a**) Dimensions of coupon; and (**b**) Failure mode of coupon.

Table 3 summarizes the vital properties of the strengthening materials utilized in the current study. For beam S-SP, steel plates were employed. These plates were attached to the compression zone using polymer adhesive (SIKA-31/41, Sika Saudi Arabia Limited, Jeddah, Saudi Arabia) in combination with high-strength threaded steel rods with a diameter of 14 mm. A total of four plates were installed: two measuring 3000 mm in length and 150 mm in width, and two measuring 1450 mm in length and 300 mm in width. All these plates had the same thickness of 25 mm. Standard coupons were prepared and tested as per relevant standards [38] for each steel plate coupon. Figure 5 depicts the details of the steel plate coupon, and Table 3 lists the test results.

**Figure 6:**
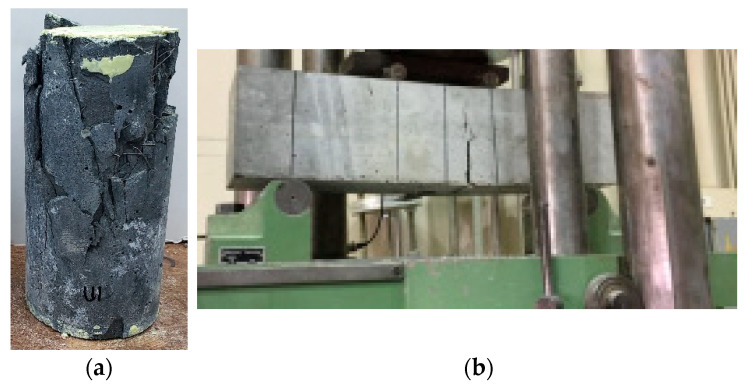
Testing of UHPFRC specimens: (**a**) Cylinder in compression test; and (**b**) Prism in flexure test.

The other strengthening material utilized in this investigation was UHPFRC, designed to achieve a compressive strength of 120 MPa. Table 4 outlines the mix proportions of the UHPFRC used. Standard specimens (prisms and cylinders) were prepared to assess their mechanical characteristics, as presented in Figure 6. The strength of UHPFRC in both flexure and compression was evaluated as per ASTM standards [37,39], and the findings are presented in Table 3. UHPFRC layer, 50 mm in thickness, was placed in the compression zone of the beam to enhance its performance. To enhance the bond at the interface between UHPFRC and the original surface, the top side of the specimen was purposefully roughened. Furthermore, 12 mm diameter hooked steel rebars were embedded into the beam to provide additional anchorage, as depicted in Figure 4.

### 2.3. Specimen Preparation

The rebar cages were assembled and inspected in compliance with the specified design requirements. Following this, the formwork was assembled, and strain gauges were installed on the rebars at predetermined locations to enable strain measurements throughout testing. Ready-mix concrete was supplied by a local provider, and to ensure consistency and minimize variability, all specimens were fabricated simultaneously. Upon curing, the specimens were prepared for strengthening. Before applying the strengthening layer, the top concrete surface was cleaned and then prepared by sandblasting to remove laitance and surface irregularities and create a rough profiled substrate essential for achieving a strong bond with the new strengthening material.

**Figure 7:**
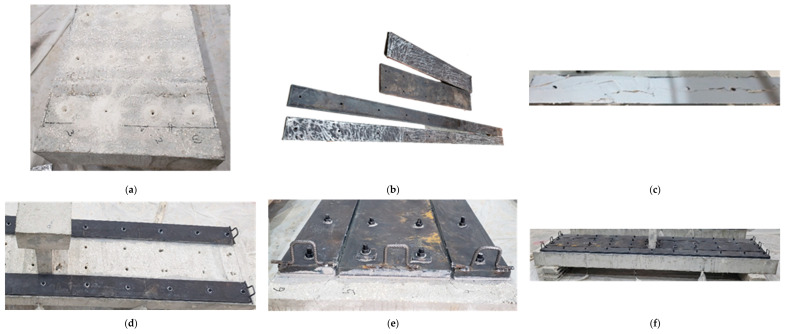
Strengthening process of specimen S-SP: (**a**) Treatment of concrete surface and drilling of holes; (**b**) Roughening of steel plate surface; (**c**) Application of Sika mortar on steel plate; (**d**) Attaching of steel plate to the beam surface; (**e**) Installation of washers and nuts; (**f**) Specimen ready for testing.

For the strengthened specimen S-SP, the hole locations were first marked on the beam and then drilled. High-strength steel rods having a 14-mm diameter were inserted in the drilled holes. To achieve full bonding, the annular space between the rods and the surrounding concrete was filled with polymer adhesive (SIKA 31/41). Corresponding holes were also drilled into the steel plates to align with the installed rods. The steel plates were then positioned over the threaded rods and pressed firmly against the concrete surface. To improve the bond at the interaction of plates and concrete, the interface surface of the plates was intentionally roughened. Adhesive mortar (SIKA 31/41) was applied to the underside of the plates to fill any voids and ensure full contact with the concrete surface. Pressure was applied to the plates until epoxy began to extrude from the interface, indicating adequate adhesion. Finally, the nuts were tightened on the threaded rods to secure the steel plates to the top surface of the beam. The configuration of the strengthened specimen is shown in Figure 3, while the strengthening procedure is illustrated in Figure 7.

**Figure 8:**
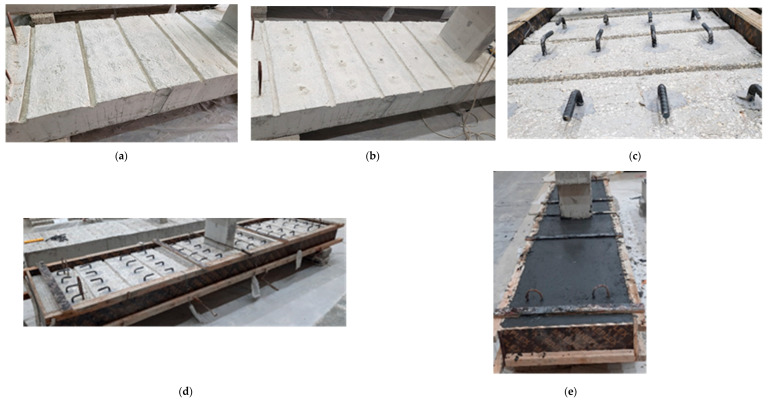
Strengthening process of specimen S-UHC: (**a**) Treatment of concrete surface and cutting of grooves; (**b**) Drilling of holes for the hooked rebars; (**c**) Installation of hooked rebars; (**d**) Installation of wooden formwork for UHPFRC casting; (**e**) Casting of UHPFRC layer.

For the strengthened beam S-UHC, transverse grooves measuring 20 × 20 mm were created on the specimen’s top surface to enhance the mechanical interlock between the UHPFRC overlay and the specimen. After that, the hole locations for the 12 mm diameter hooked rebars were marked and drilled. The rebars were then inserted into the predrilled holes, with a 100 mm embedment length into the existing concrete. The void around the rebars and surrounding concrete was filled with adhesive mortar (SIKA 31/41) to ensure a secure bond. These rebars were hooked into the UHPFRC layer to further strengthen its bond with the beam. Once the epoxy mortar was fully cured, formwork was installed to accommodate casting of the UHPFRC overlay. The UHPFRC mix was fabricated in the laboratory, and it was subsequently cast onto the top face of the wide beam. Figure 4 provides details of the retrofitted specimen, while Figure 8 presents the strengthening process.

### 2.4. Instrumentation and Testing

**Figure 9:**
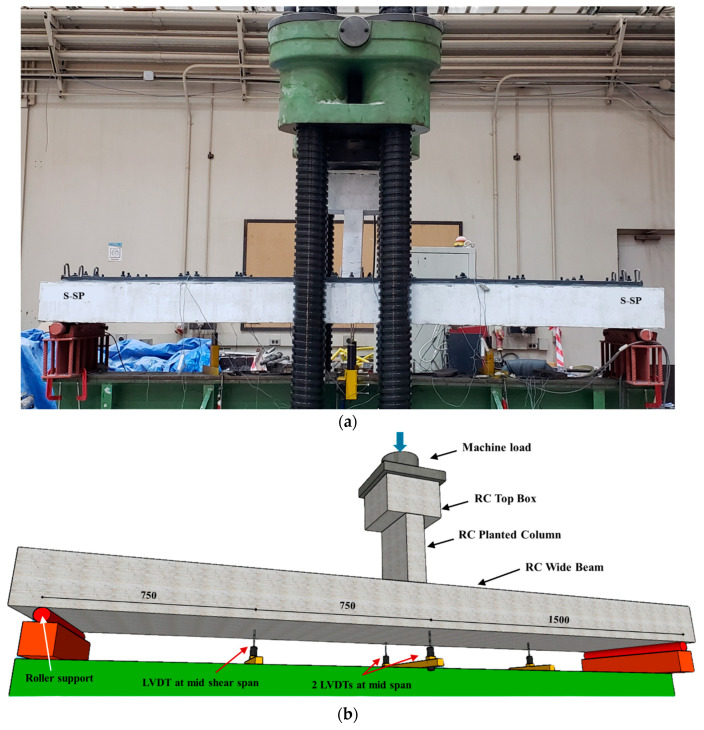
Test set up and instrumentation layout (**a**) S-SP ready for test; (**b**) Isometric view (Units: mm).

As shown in Figure 9, the specimens were subjected to single-point bending tests utilizing an AMSLER testing apparatus (Alfred J. AMSLER & Co., Schaffhouse, Switzerland) with a maximum capacity of 2000 kN. The machine’s in-built load cell was used to record load data while a displacement-controlled loading protocol was used at a steady rate of 2 mm/min. The planted column at the mid-span directly transmitted the applied load. With shear spans of 1500 mm on either side, each simply supported specimen had a clear span of 3000 mm. Figure 9 illustrates the experimental setup, including the arrangement of instrumentation used during testing. Mid-span displacements were monitored utilizing two LVDTs, while an additional LVDT was positioned in every shear span to record the beam deformation. Additionally, strain gauges were affixed to capture strain at several critical locations. For all tested specimens, strain gauges were installed at the mid-span on both the longitudinal bottom and top rebars. Strain gauges were also placed on selected steel stirrups within the shear span to monitor shear behavior. For the strengthened specimens, strain gauges were applied to the strengthening materials. For specimen S-SP, strain gauges were attached to the steel plates at both mid-span and middle of shear span. For specimen S-UHC, strain gauges were attached to the top surface of the UHPFRC layer at mid-span to record the maximum compressive strain in the overlay.

## 3. Discussion of Experimental Findings

### 3.1. Failure Pattern and Peak Load

**Figure 10:**
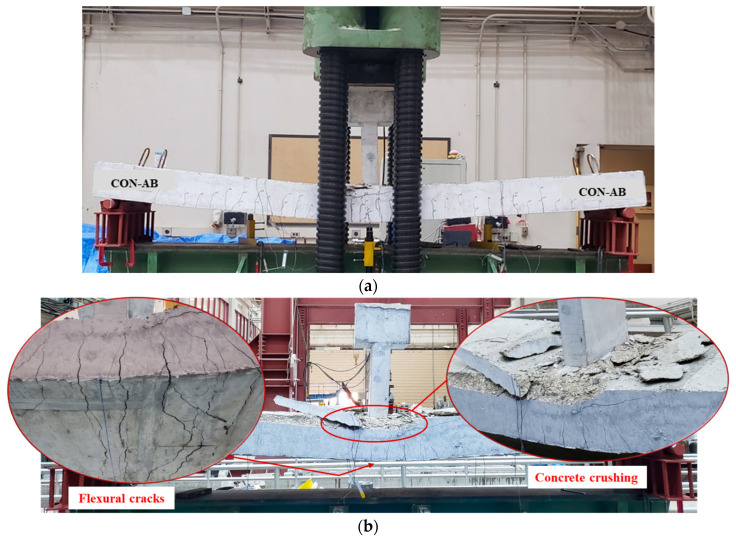
Failure mode of reference beam CON-AB: (**a**) Ultimate failure mode; (**b**) Close-up view identifying flexural cracking and concrete crushing.

**Figure 11:**
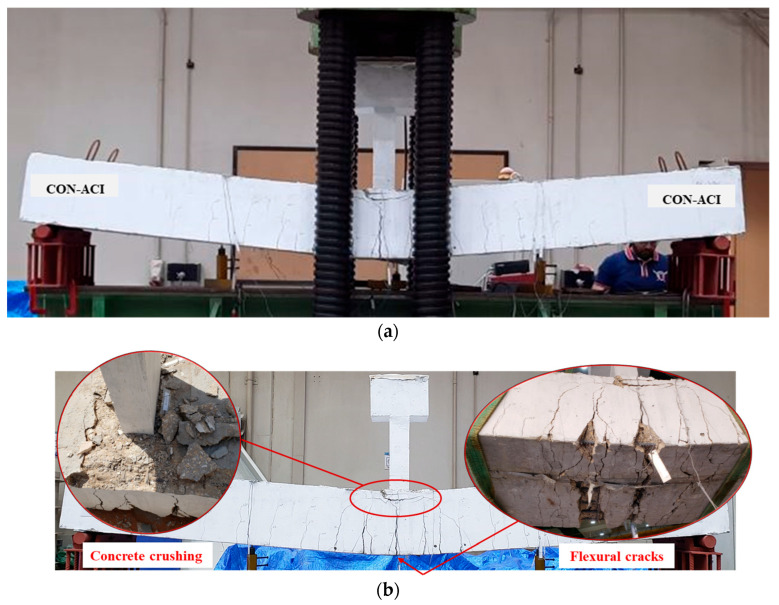
Failure mode of reference beam CON-ACI: (**a**) Ultimate failure mode; (**b**) Close-up view identifying flexural cracking and concrete crushing.

Table 5 depicts the vital test findings for the specimens, incorporating the total cracking load and corresponding deflection at mid-span, the yield load that corresponded to the onset of yield in bottom rebars and related deflection, the ultimate load and its related deflection, as well as the observed failure modes. Both control specimens, CON-AB and CON-ACI, exhibited identical failure behavior. As shown in Figure 10 and Figure 11, both beams failed in a typical flexural mode characteristic of under-reinforced, tension-controlled reinforced concrete members. Prominent flexural cracks developed at mid-span and became visible upon yielding of the tensile steel reinforcement. These cracks extended nearly vertically toward the top face. At larger deflections, concrete crushing was noted in the top zone near the planted column. The peak loads of the two un-retrofitted specimens were nearly identical, which is consistent with their design, as both were intended to possess the same flexural strength.

**Figure 12:**
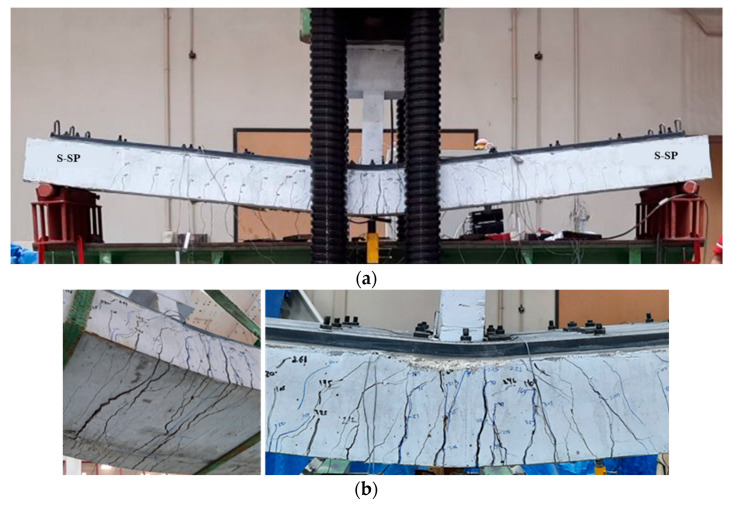
Failure mode of upgraded beam S-SP: (**a**) Ultimate failure mode; (**b**) Bottom and side concrete surface showing flexural cracking.

The strengthened beam S-SP exhibited a flexural mode of failure. The first major flexural crack appeared near the mid-span, as presented in Figure 12. When the load increased, a corresponding increase in deflection was noticed. Flexural cracking progressed further on the tension side, concentrated around the middle zone. Eventually, failure was noticed in the bottom concrete following the yielding of the tensile reinforcement. Importantly, no localized failure, such as concrete splitting or pull-out, was observed around the anchoring rods, confirming that the anchorage system was not a limiting factor in the failure. This sequence confirms a typical flexural failure mechanism characterized by post-yield deformation prior to ultimate failure.

**Figure 13:**
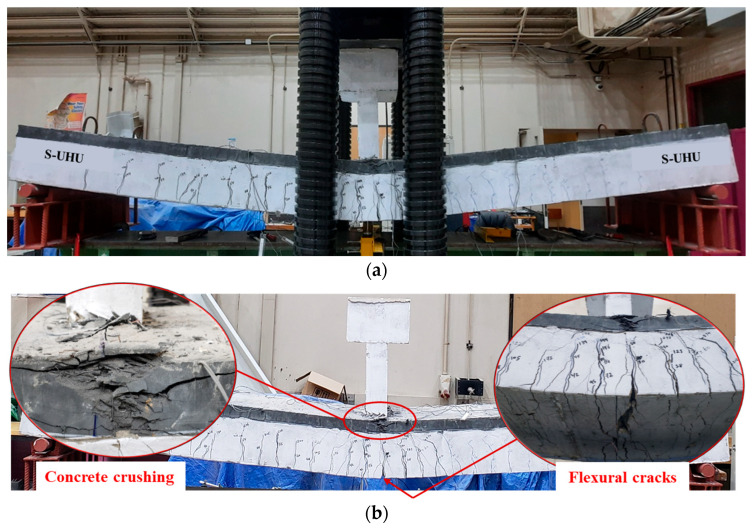
Failure mode of upgraded beam S-UHC: (**a**) Ultimate failure mode; and (**b**) Close-up view identifying flexural cracking and UHPFRC crushing.

The strengthened beam S-UHC also exhibited a flexural mode of failure. The first vertical crack was noticed near the beam mid-span, as illustrated in Figure 13. When the load increased, a corresponding rise in mid-span deflection was recorded, accompanied by propagation of additional vertical cracks on the tension side. Eventually, the bottom concrete experienced failure following the yielding of tension bars. This was associated with localized crushing of the UHPFRC in the compression region, as shown in Figure 13. Notably, no delamination or separation occurred between the UHPFRC overlay and the original beam, and no local failures were observed at the hooked rebar locations, indicating the effectiveness of the entire bonding and anchorage technique employed in this strengthening scheme.

Overall, the application of both steel plates and UHPFRC was evidenced to be efficient in enhancing the ultimate load of RC shallow beams. For specimen S-SP, the use of steel plates on the top face caused a 69% enhancement in ultimate load with respect to the unstrengthened beams. Similarly, the inclusion of UHPFRC overlay on the compression side of specimen S-UHC caused a 71% increase in ultimate load capacity. These results highlight the effectiveness of both schemes in significantly upgrading the structural performance of RC shallow beams.

### 3.2. Load–Deflection Behavior

**Figure 14:**
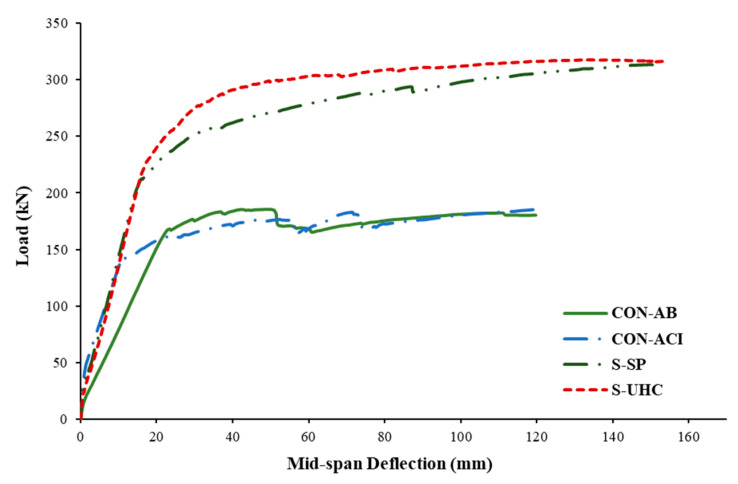
Relationship of load versus middle deflection of shallow beams.

Figure 14 illustrates the load–deflection responses at mid-span for all beams. Both control specimens (CON-AB and CON-ACI) exhibited typical trilinear behavior characteristic of tension-controlled RC beams. The preliminary linear portion was owing to the uncracked phase, noticeable by high stiffness. Once concrete cracking occurred, the stiffness decreased, forming the second segment of the curve, which continued until the yielding of the bottom bars. The third portion of the curve represents the post-yielding phase, where a further reduction in stiffness is observed, creating the yielding plateau.

Among the control specimens, CON-ACI demonstrated substantially higher stiffness throughout the test with regard to CON-AB. This enhanced performance is because CON-ACI was designed to satisfy serviceability requirements, specifically the deflection limits stipulated by relevant design codes. In contrast, CON-AB was representative of existing wide beams with deflections exceeding these limits.

Both strengthened specimens, S-SP and S-UHC, exhibited noticeably increased stiffness with regard to the reference specimen CON-AB. The effectiveness of the strengthening techniques in reducing deflection and improving overall structural performance was demonstrated by the fact that their stiffness performance was roughly equal to that of the code-compliant control beam CON-ACI.

As demonstrated in Figure 14, both retrofitted beams (S-SP and S-UHC) displayed a clearer yielding plateau when compared with the control specimens. This behavior reflects the enhanced ductility resulting from the incorporation of steel plates and the UHPFRC layer. The load–deflection responses presented in Figure 14 and the corresponding values in Table 5 confirm that the addition of strengthening materials at the compression side, steel plates in specimen S-SP and UHPFRC in specimen S-UHC, significantly improved the structural performance when compared with the reference beam CON-AB.

In specimen S-SP, the addition of steel plates upgraded the yield and ultimate loads by approximately 23% and 69%, respectively. However, it also resulted in a 34% reduction in yield deflection, indicating a stiffer behavior prior to yielding. Similarly, in specimen S-UHC, the application of a UHPFRC layer increased yield and ultimate loads by 34% and 71%, respectively, while decreasing the yield deflection by 25%.

When compared to the code-compliant beam CON-ACI, the retrofitted beams exhibited even greater gains in yield load, 26% for S-SP and 37% for S-UHC. However, the improvements in ultimate load for both strengthened specimens remained nearly identical to those observed relative to the non-compliant reference beam CON-AB. These results demonstrate how both strengthening methods can improve serviceability performance by increasing stiffness and decreasing deflection while also increasing strength and ductility.

Table 5 summarizes the experimentally obtained values of specimen stiffness at different stages (pre-cracking, secant, and post-cracking), dissipated energy, and displacement ductility. The key parameters used to derive these values were determined as follows. The cracking load (*P_cr_*) was identified as that corresponding to the first concrete cracking. The first cracking deflection (Δ*_cr_*) is the mid-span deflection associated with *P_cr_*. The yield load (*P_y_*) was identified as the load at which the strain gauges on the main tensile reinforcement at mid-span first reached the yield strain. The yield deflection (Δ*_y_*) is the mid-span deflection corresponding to *P_y_*. The ultimate deflection (Δ*_pu_*) was defined as the mid-span deflection recorded at the maximum load (*P_u_*). Based on these points, the stiffness at the pre-cracking phase (*k_un_*) was evaluated as the first cracking load (*P_cr_*) divided by the associated deflection (Δ*_cr_*). The stiffness at the post-cracking phase (*k_po_*) is the slope of the load–deflection plot between the cracking and yield points. The secant stiffness (*k_s_*) was assessed as the ratio between the yield load (*P_y_*) and the associated yield deflection (Δ*_y_*). The dissipated energy (*E_u_*) was quantified as the area under the load–deflection plot up to failure, indicating the specimen’s energy absorption capacity [24,34]. Finally, the displacement ductility (*μ*_Δ_) was defined as the ratio of the deflection at failure to the yield deflection (Δ*_y_*), reflecting the beam’s deformation capacity beyond the elastic limit [24,34].

It should be mentioned that, when compared to the control specimen CON-AB, the retrofitted beam S-SP’s flexural stiffness increased by 86%. Furthermore, its stiffness was 7% higher than that of the CON-ACI reference specimen. Additionally, the test findings showed that, in comparison to the control specimens CON-AB and CON-ACI, the dissipated energy rose by 113% and 106%, respectively. Furthermore, as compared to control specimens CON-AB and CON-ACI, respectively, the retrofitted beam S-SP demonstrated an improvement in deflection ductility of 93% and 7%, in turn.

The flexural stiffness of the retrofitted specimen S-UHC was 80% higher than that of the unstrengthened beam CON-AB. Furthermore, its stiffness was 3% higher than that of the CON-ACI reference specimen. Furthermore, in comparison to the unstrengthened beams CON-AB and CON-ACI, the test findings showed a dissipated energy increase of 126% and 118%, respectively. Additionally, the displacement ductility of the retrofitted beam S-UHC was 72% higher than that of the control specimen CON-AB, but it was 5% lower than that of the ideal control beam CON-ACI.

### 3.3. Load–Strain Characteristics

**Figure 15:**
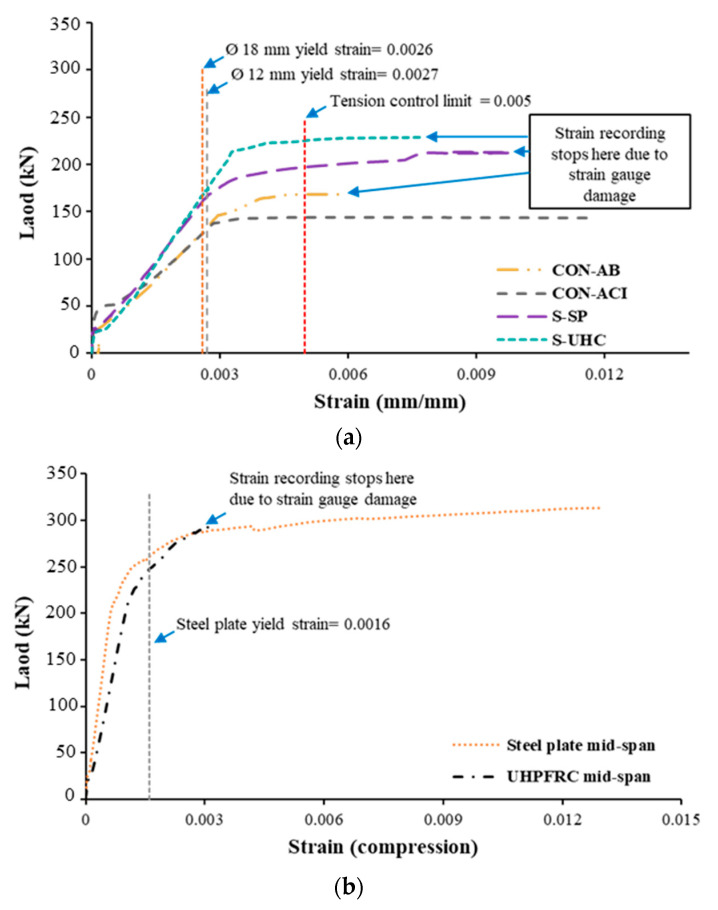
Load versus mid-span strain in: (**a**) Main steel rebars; (**b**) Steel plate and UHPFRC layer (compression).

Figure 15a presents the load versus strain curves of the main rebars at mid-span for the four tested beams. As shown, all specimens satisfied the tension-controlled criterion specified by current design codes, with maximum recorded strains significantly exceeding 0.5%. This indicates that the tensile rebars yielded before failure. Figure 15b presents the load versus strain relationships at mid-span for the steel plate and UHPFRC layer in the strengthened beams S-SP and S-UHC, in turn. The findings reveal that the steel plates in specimen S-SP yielded in compression, confirming their effective role in enhancing both flexural capacity and stiffness. For specimen S-UHC, the UHPFRC layer reached a maximum compressive strain of about 0.0135, demonstrating not only improved stiffness but also the ductile nature of the specimen’s overall response.

### 3.4. Comparative Assessment of Strengthening Systems

**Figure 16:**
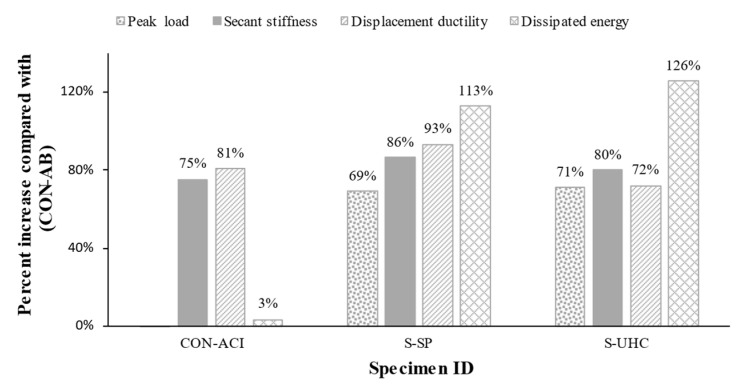
Percent increase of evaluated parameters compared with control specimen CON-AB.

Both upgraded beams demonstrated superior flexural performance with respect to the unstrengthened beams. As illustrated in Figure 14, the strengthened beams exhibited increased load capacity, enhanced stiffness, and improved ductility due to the incorporation of steel plates and UHPFRC as strengthening materials. Figure 16 presents the percentage increase in key performance parameters of the tested specimens relative to the reference specimen CON-AB. The results in Figure 16 further validate the efficacy of the adopted retrofitting techniques, showing that the retrofitted beams not only significantly improved the structural performance but also exhibited lower deflections than the ideal control specimen CON-ACI, thereby meeting serviceability requirements more effectively. As illustrated in Figure 16*,* with respect to reference beam CON-AB, the secant stiffness increased by 75%, 86%, and 80% for specimens CON-ACI, S-SP, and S-UHC, respectively.

Also, the displacement ductility increased by 81%, 93%, and 72% for specimens CON-ACI, S-SP, and S-UHC, respectively, compared with the control beam CON-AB. The dissipated energy increased, respectively, by 113% and 126% for strengthened specimens S-SP and S-UHC; however, it only increased by 3% for the control specimen CON-ACI. It is also identified from Table 5 that with respect to the un-retrofitted specimen CON-AB, the deflection at yield load decreased by 34% and 25% for specimens S-SP, and S-UHC, in turn. These findings indicate that both strengthening schemes were effective in upgrading shallow beams having planted columns to satisfy the serviceability requirements of current codes. Notably, the proposed strengthening techniques not only enabled compliance with deflection limits but also provided superior load–deflection performance with respect to the code-compliant reference beam CON-ACI.

Beyond their quantitative performance, both systems offer distinct practical advantages. The steel plate system utilizes conventional, widely available materials, and its installation is a robust mechanical bolting process that ensures a predictable and stiff composite action. The UHPFRC system provides a lightweight strengthening solution that can also be a more economical alternative. Its cast-in-place nature offers excellent adaptability for irregular beam geometries, and its installation method, involving thorough substrate preparation, is designed to achieve a monolithic and highly effective bond with the original beam. This highlights that both are viable, high-performance techniques, allowing for an informed choice based on project-specific needs.

The current work focused on the short-term performance of RC shallow beams strengthened on the compression side using bonded steel plates or UHPFRC overlays, with particular emphasis on reducing instantaneous deflection and enhancing load capacity. Long-term effects, including creep, shrinkage, and temperature variations, as well as interface durability between the original concrete and the strengthening materials, were beyond the present scope. Nevertheless, these factors can significantly influence the time-dependent deformation and overall durability of strengthened members and are therefore recommended for future investigation.

## 4. Analytical Investigation

**Figure 17:**
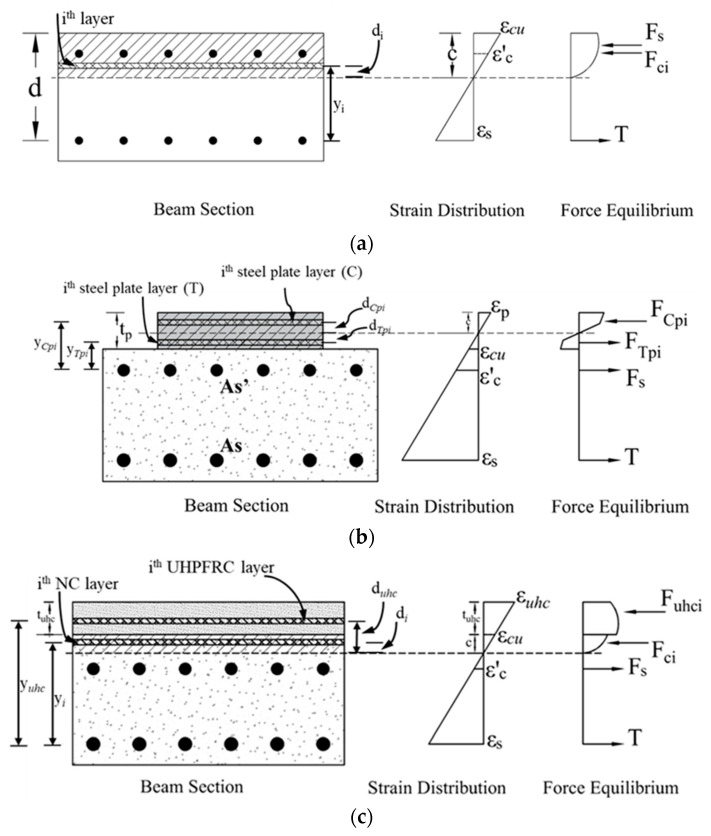
Internal stress and strain distribution for: (**a**) Reference beams; (**b**) S-SP; (**c**) S-UHC.

**Figure 18:**
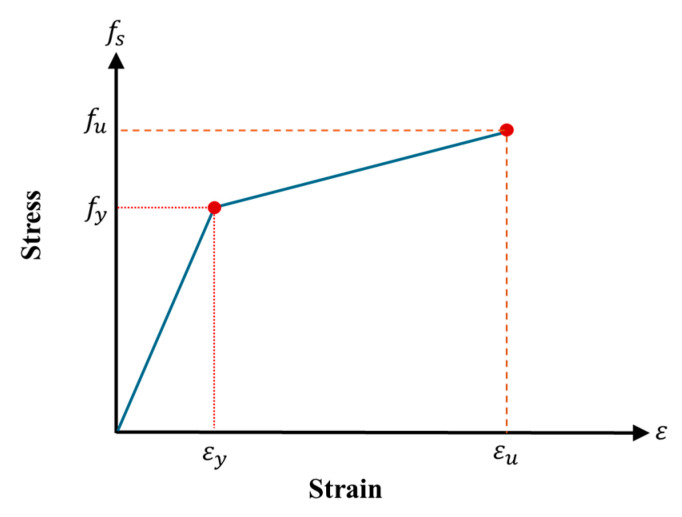
Stress–strain curve for reinforcing steel.

With the help of the laminar analysis approach, the shallow beam section was subdivided into several thin layers, as depicted in Figure 17. Stress calculations for each layer were performed using Mander’s model [40] for normal concrete and Naeimi’s model [41] for UHPFRC. The stresses in the steel plates, as well as in the tensile and compressive reinforcement, were evaluated utilizing a bilinear stress–strain model, illustrated in Figure 18.

An iterative procedure was applied to determine the depth of the neutral axis, ensuring equilibrium between internal tensile and compressive forces contributed by the concrete, steel reinforcement, steel plates, and UHPFRC layers. It is worth mentioning that the concrete’s tensile strength was disregarded, and the ultimate concrete compressive strain was taken as *ε_cu_* = 0.003. The internal force equilibrium equation for specimen S-UHC is given as:

(3) $$F_{ci}+F_{uhci}={T+F}_{s}$$

where $F_{Ci}\;and\;F_{uhci}$ are the compression forces in normal concrete and UHPFRC, respectively, and T and $F_{s}$ are, in turn, the tension forces in bottom and top steel rebars. These forces can be calculated as

(4) $$F_{Ci}=A_{i}f_{ci}\;\&\;F_{uhci}=A_{uhci}f_{uhci};\;T=A_{s}f_{s}\;\&\;F_{s}={A^{\prime }}_{s}{f^{\prime }}_{s}$$

where $A_{i}\;\&$ $f_{ci}$ are area and stress of the *i*th layer of normal concrete; $A_{uhci}$ $\&\;\;f_{uhci\;}$ are area and stress of each the *i*th layer of UHPFRC material; $A_{s}{\;\&\;f}_{s}$ are area and stress of bottom rebars; and ${A^{\prime }}_{s}\;\&\;{f^{\prime }}_{s}$ are area and stress of the rebars. The stresses in the above equation were calculated from the strains in each material, which were computed from

(5) $$\varepsilon_{ci}=0.003\left(\frac{d_{ci}}{c}\right){\&\;\varepsilon }_{uhci}=0.003\left(\frac{d_{uhci}}{c}\right);\;\varepsilon_{s}=0.003\left(\frac{d-c}{c}\right)\;\&\;{\varepsilon^{\prime }}_{s}=0.003\left(\frac{d^{\prime }-t_{uhc}-c}{c}\right)$$

where $\varepsilon_{ci}$ is the strain in each normal concrete layer; $\varepsilon_{uhci}$ is the strain in each UHPFRC layer; ${\varepsilon_{s}\;\&\;\varepsilon^{\prime }}_{s}$ are the strain in tensile and compression reinforcement, respectively; $d_{ci}$ is the distance from the neutral axis to the concrete layer; $d_{uhci}$ is the distance from the neutral axis to the UHPFRC layer; *d* is the distance from the center of tension bars to the beam top fiber; $d^{\prime }$ is the distance from the center of compression bars to the extreme compression fiber; and *c* is neutral axis depth.

After fulfilling the internal forces’ equilibrium in Equation (3), the bending resistance of the beam was computed from

(6) $$M_{n}=\sum_{i}^{n}F_{uhci}Y_{uhci}+\sum_{i}^{n}F_{ci}Y_{ci}+F_{s}(d+t_{uhc}-d^{\prime })$$

where $Y_{uhci}$ is the distance from the tension bars to the *i*th layer of UHPFRC material; $Y_{ci}$ is the distance from the tensile rebars to the concrete layer; and $t_{uhc}$ is the total thickness of UHPFRC overlay.

**Figure 19:**
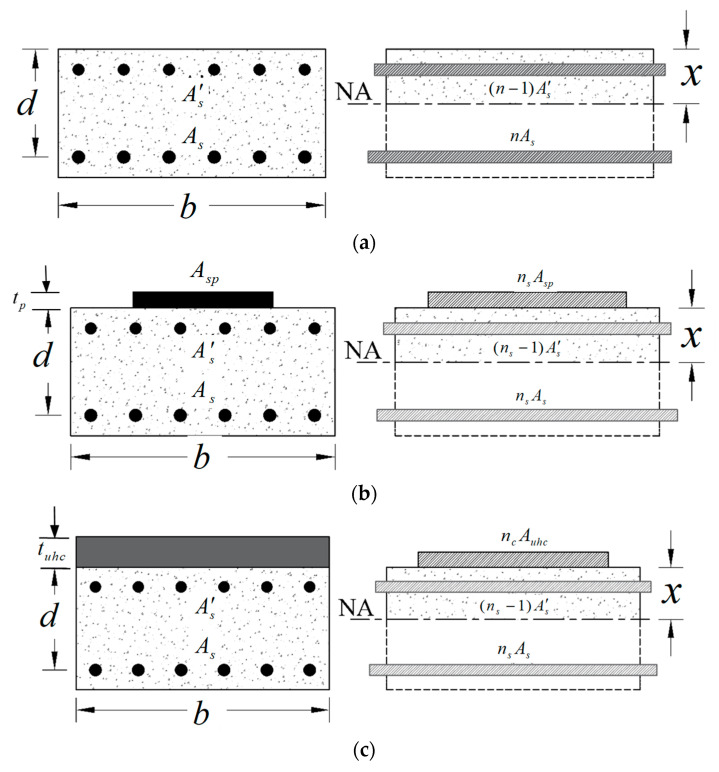
Transformed section for: (**a**) Reference beams; (**b**) S-SP; (**c**) S-UHC.

In current research, deflection estimates adhered to the methodology suggested by ACI [35,42]. The gross moment of inertia (*I_g_*) is appropriate for expressing flexural stiffness and calculating instantaneous deflections for prismatic RC beams that are not cracked. On the other hand, in order to obtain precise and dependable deflection estimations for cracked sections, the effective moment of inertia (*I_e_*) should be used and computed as

(7) $$I_{e}={\left(\frac{M_{cr}}{M_{a}}\right)}^{3}I_{g}+\left[1-{\left(\frac{M_{cr}}{M_{a}}\right)}^{3}\right]I_{cr}\leq I_{g}$$

where *I_cr_* stands for the cracking inertia of the transformed section (see Figure 19); *M_a_* is the moment at the state during which deflection is assessed; and *M_cr_* is the cracking moment, evaluated from Ref. [35] as follows:

(8) $$M_{cr}=\frac{f_{r}I_{g}}{y_{t}}$$

where *f*_r_ stands for concrete’s modulus of rupture; and *y_t_* is the gross section centroid to the extreme tension fiber.

For developing the analytical load–deflection graphs of the tested specimens, the load was raised gradually in increments of 0.7 kN, beginning at zero. At each step, the moment *M_a_* was computed and then the effective moment of inertia *I_e_* was estimated. Next, the simply supported beams’s deflection δ under a middle point load was computed from

(9) $$\delta =\frac{PL^{3}}{48E_{c}I_{e}}$$

where *P* is the middle point load; *L* is the beam span; and *E_c_* is the elastic modulus of concrete, given from Ref. [35] as

(10) $$E_{c}=4700\sqrt{{f^{\prime }}_{c}}$$

where ${f^{\prime }}_{c}$ is the concrete’s compressive strength (in MPa).

The total mid-span deflection, including long-term effects, was computed as per ACI [42] from

(11) $$\delta_{total}=\delta_{L}+\lambda \;\delta_{D+SL}$$

In the above equation, *λ* is a multiplier accounting for long-term effects; and *δ_D_*_+*SL*_ is the mid-span deflection owing to dead and sustained live loads. In the current study, the sustained live load was assumed as 25% of the design live load (assuming residential buildings); and *δ_L_* is the live load deflection given by

(12) $$\delta_{L}=\delta_{D+L}-\delta_{D}$$

where $\delta_{D+L}\;\&\;\delta_{D}$ are, respectively, the instantaneous deflections due to dead plus live loads and dead load only. The predicted total deflection was computed as follows:

After calculating the beam’s flexural resistance (*M_u_*), the maximum load was calculated. The dead and live loads were then assessed by assuming the live load as a proportion of the dead load. Then, deflections were evaluated under three loading conditions: dead load ($\delta_{D}$), combined dead and live loads ($\delta_{D+L}$), and dead plus sustained live loads ($\delta_{D+SL}$), as determined from Equations (11) and (12). The multiplier λ that accounts for long-term effects was computed according to the following formula.

(13) $$\lambda =\frac{\xi }{1+50\rho^{\prime }}$$

In Equation (13), ξ is a time-dependent code parameter accounting for sustained loads; and $\rho^{\prime }$ is the ratio of compression steel.

Based on the test results, the total deflection accounting for long-term effects was also computed using Equations (11) and (12). Nevertheless, the deflections at dead load (*δ_D_*), combined dead and live loads ($\delta_{D+L}$), and dead plus sustained live loads ($\delta_{D+SL}$) were extracted from the experimental load versus deflection curves.

### Discussion of Analytical Results

**Figure 20:**
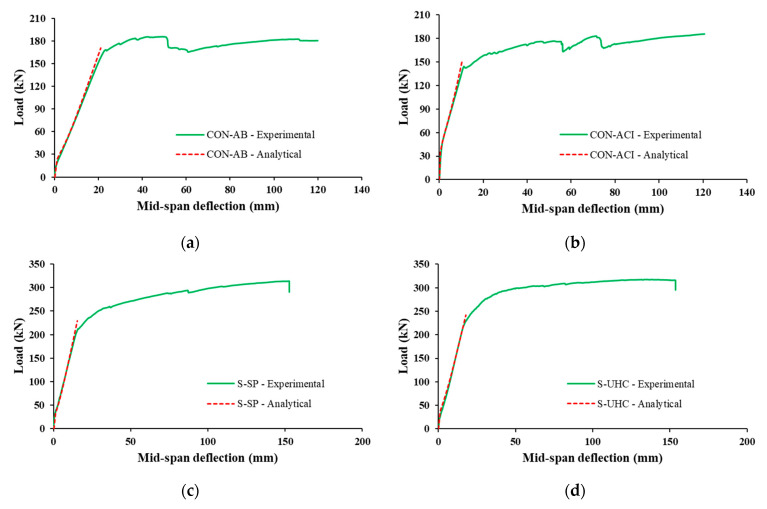
Comparison of measured and predicted load–deflection plots for: (**a**) CON-AB; (**b**) CON-ACI; (**c**) S-SP; (**d**) S-UHC.

A thorough comparison of the experimental and analytical findings for the tested specimens’ peak load, secant stiffness, and total deflection is shown in Table 6. It should be noted that, after taking long-term effects into account, the total deflections shown in Table 6 are the sum of the instantaneous deflection brought on by live load and the additional deflection brought on by dead and sustained live loads. Additionally, a comparison of the experimentally measured and analytically predicted load–displacement responses is shown in Figure 20, focusing on the immediate deflection behavior of the specimens.

The comparison indicates that the analytical model provided reasonably accurate predictions for peak load, secant stiffness, and total deflection for both control and strengthened specimens. As demonstrated in Table 6, the error in predicting the peak load was within 7%, while the prediction errors for secant stiffness reached up to 9%. For total deflection, the analytical predictions showed errors up to 8%.

**Figure 21:**
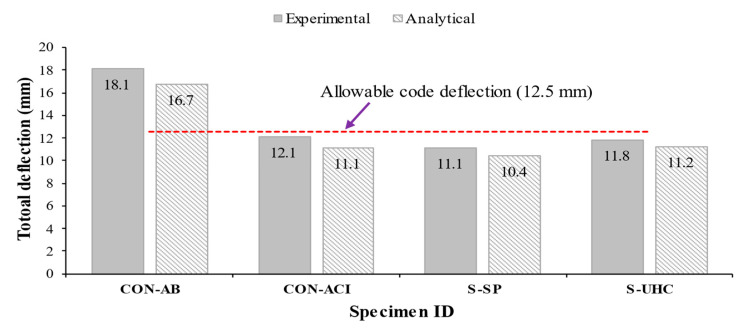
Measured versus predicted total deflection of shallow beams.

Figure 21 compares the experimental and predicted total deflections of the tested beams. It is clear that specimens CON-ACI, S-SP, and S-UHC satisfied the serviceability requirements of the design code, exhibiting deflection values lower than the allowable limit of 12.5 mm (L/240). In contrast, the as-built control specimen CON-AB exceeded this threshold, confirming the need for strengthening.

## 5. Conclusions

This study presents the testing campaign of shallow beams having planted columns, strengthened in flexure to control deflection using either steel plates or a UHPFRC layer added on the compression region. The testing findings were discussed pertaining to failure patterns and load–mid-span deflection response. Furthermore, the experimental findings were compared with analytical predictions for both deflection and flexural capacity of the beams. The main findings of this research are summarized as follows:

1. The common flexural failure mode of tension-controlled beams was present in both the control and retrofitted specimens. It involved the development of the main vertical cracks in the middle of the span, the tension bars’ yielding, and the concrete’s ultimate crushing on the compression side next to the planted column.
2. The two strengthening schemes, steel plates and UHPFRC layer, proved highly efficient in upgrading the flexural response of shallow beams. Both methods significantly improved strength and stiffness while reducing total deflection to within serviceability limits prescribed by current design codes.
3. Both the steel plate and UHPFRC overlay techniques proved highly effective, yielding comparable and substantial enhancements relative to the deficient control beam. Compared with the control specimen (CON-AB), both strengthening systems markedly increased the maximum load capacity, flexural stiffness, displacement ductility, and energy dissipation. Moreover, both upgraded beams successfully satisfied the serviceability (deflection) criteria, exhibiting superior performance to the code-compliant reference beam (CON-ACI).
4. The analytical models developed in this study provided reasonably accurate predictions for the structural performance of both control and strengthened RC wide beam specimens. The errors in computing the maximum load were within 7%, for secant stiffness within 9%, and for total deflection within 8%. Moreover, for the immediate deflection, a strong correlation was observed between the predicted and experimentally measured load–deflection curves, further confirming the reliability of the developed models in accurately representing both the flexural behavior and serviceability performance of the tested beams.
5. The findings of current research are limited to the case of simply supported RC wide beams and cannot be extended to continuous RC shallow beams. Also, the strengthening systems used in this study are added to the top surface of the RC wide beam. In cases where the top side of the beam is inaccessible, these techniques may not be feasible, and other upgrading techniques that may be added on the tension side could be more relevant.
6. Compared with the authors’ earlier work on tension-side strengthening, which improved stiffness but caused brittle debonding and reduced ductility, the present compression-side schemes markedly enhanced strength, stiffness, and displacement ductility. Therefore, compression-side strengthening is recommended for effective deflection control in RC shallow beams with planted columns. Future studies should explore alternative systems for cases where the compression face is inaccessible to ensure ductile performance without debonding failures.
7. A main limitation of this study is that only one specimen was tested for each configuration, which prevents statistical assessment of variability. Although the observed responses are consistent with established trends, the limited sample size constrains the generality of the findings. Future work should include replicates and parametric variations to enhance statistical reliability. Moreover, incorporating full-scale testing or finite element modeling would complement the experimental results and allow a broader parametric exploration of RC shallow beams strengthened in flexure using steel plates or UHPFRC overlay added on the compression side.

## Figures and Tables

**Table 1:** Summary of test specimens.

Specimen ID	Details
**CON-AB**	Unstrengthened reference beam Fulfills requirements of design codes with respect to flexure and shear Does not fulfill requirements of design codes with respect to deflection Total deflection (accounting for long-term) = 16.7 mm > codified threshold (=3000/240 = 12.5 mm)
**CON-ACI**	Unstrengthened reference beam Fulfills requirements of design codes with respect to flexure and shear Fulfill requirements of design codes with respect to deflection Total deflection (accounting for long-term) = 11.1 mm < codified threshold (=3000/240 = 12.5 mm)
**S-SP**	Retrofitted beam Has dimensions and reinforcement similar to reference beam CON-AB, but it was retrofitted using steel plates added on the compression side. Total deflection (accounting for long-term) = 10.4 mm < codified threshold (=3000/240 = 12.5 mm)
**S-UHC**	Retrofitted beam Has dimensions and reinforcement similar to reference beam CON-AB, but it was retrofitted using UHPFRC overlay added on the compression side. Total deflection (accounting for long-term) = 11.2 mm < codified threshold (=3000/240 = 12.5 mm)

**Table 2:** Computation of specimens’ deflection *.

Specimen	*δ_D_* (mm)	*δ_D_*_+*L*_ (mm)	*δ_L_* (mm)	*δ_D_*_+*SL*_ (mm)	*δ_total_ = δ_L_ + λδ_D_*_+*SL*_ (mm)	Check Deflection (Allowable = 12.5 mm)
CON-AB	9.3	13.4	4.1	10.4	16.7	Exceeds
CON-ACI	5.9	8.5	2.6	6.6	11.1	Satisfies
S-SP	5.7	8.3	2.5	6.4	10.4	Satisfies
S-UHC	6.3	9.0	2.7	7.0	11.2	Satisfies

* δ_D_: dead load deflection; *δ_D_*_+*L*_: deflection owing to dead and live loads; *δ_L_*: live load deflection; *δ_D_*_+*SL*_: deflection due to dead and sustained live loads; *δ_total_*: total deflection; *λ =* long-term multiplier [31].

**Table 3:** Material properties.

Parameter	Properties
*Concrete*	
Compressive strength on testing day	40 MPa
*UHPFRC*	
Compressive strength on testing day	120 MPa
Flexural strength	19.5 MPa
*Steel Rebars*	
Diameter (mm)	Yield (ultimate) strength (MPa)
8	525 (537)
10	547 (572)
12	550 (657)
18	528 (658)
*Steel Plate*	
Elastic modulus (MPa)	200,000 MPa
Yield (ultimate) strength (MPa)	330 (492)
*Polymer Adhesive Mortar* *	
Compressive (tensile) strength (MPa)	52 (13) MPa
Elastic modulus	2600 MPa
Bond strength to concrete	>4 MPa

* Based on manufacturer’s datasheet.

**Table 4:** Mix design of UHPFRC used in current study.

Material	Value
Cement OPC (kg/m^3^)	900
Sand (kg/m^3^)	990
Water (kg/m^3^)	195
Micro-silica (kg/m^3^)	222
Super plasticizer: Master Glenium 51 (kg/m^3^)	26
Hooked end steel fibers 1% (kg/m^3^)	79
Water to binder ratio	0.22

**Table 5:** Experimental findings of shallow beams *.

Specimen ID	*P_cr_* (kN)	Δ*_cr_* (mm)	*P_y_* (kN)	Δ*_y_* (mm)	*P_u_* (kN)	Δ*_pu_* (mm)	*k_un_* (kN/mm)	*k_s_* (kN/mm)	*k_po_* (kN/mm)	*E_u_* (kN.m)	*μ*_Δ_	Mode of Failure
CON-AB	21.4	1.6	140.7	18.5	185.4	49.6	13.4	7.6	7.1	19.2	6.5	FY-C
CON-ACI	54.9	2.0	137.7	10.3	185.3	71.4	27.3	13.3	10.0	19.9	11.7	FY-C
S-SP	38.8	1.5	173.0	12.2	313.5	152.7	25.7	14.2	12.6	40.9	12.5	FY
S-UHC	25.9	1.0	189.0	13.8	317.4	134.1	26.0	13.7	12.7	43.4	11.2	FY-UC

* *P_cr_*: load at first concrete cracking; *P_y_*: load at first yield of tension rebars; *P_u_*: peak load; Δ*_cr_*: mid-span deflection at *P_cr_*; Δ*_y_*: mid-span deflection at *P_y_*; Δ*_pu_*: mid-span deflection at *P_u_*; *k_un_*: stiffness in the uncracked phase; *k_s_*: secant stiffness; *k_po_*: stiffness in the post-cracking phase; *E_u_*: dissipated energy; *μ*_Δ_: displacement ductility; FY-C: flexural cracking and yielding of bottom rebars succeeded by concrete crushing; FY: flexural cracking and yielding of bottom rebars; FY-UC: flexural cracking and yielding of bottom rebars succeeded by UHPFRC crushing.

**Table 6:** Comparison of experimental and analytical findings of shallow beams *.

Specimen ID	*P_u,Exp_* (kN)	*P_u,Anl_* (kN)	*P_u,Exp_*/*P_u,Anl_*	*k_s,Exp_* (kN/mm)	*k_s,Anl_* (kN/mm)	*k_s,Exp_*/*k_s,Anl_*	Δ*_t,Exp_* (mm)	Δ*_t,Anl_* (mm)	Δ*_t,Exp_/*Δ*_t,Anl_*
CON-AB	185.4	173.3	1.07	7.6	8.1	0.94	18.1	16.7	1.08
CON-ACI	185.3	194.5	0.95	13.3	14.6	0.91	12.1	11.1	1.08
S-SP	313.5	305.3	1.03	14.2	14.8	1.96	11.1	10.4	1.07
S-UHC	317.4	301.3	1.05	13.7	13.7	1.00	11.8	11.2	1.05

* *P_u,Exp_*: Ultimate experimental load; *P_u,Anl_*: Ultimate analytical load; *k_s,Exp_*: Experimental secant stiffness; *k_s,Anl_*: Analytical secant stiffness; Δ*_t,Exp_*: Experimental total deflection; Δ*_t,Anl_*: Analytical total deflection.

## Data Availability

The raw data supporting the conclusions of this article will be made available by the authors on request.
